# Processes and outcomes of diabetes mellitus care by different types of team primary care models

**DOI:** 10.1371/journal.pone.0241516

**Published:** 2020-11-05

**Authors:** Fangjian Guo, Yu-Li Lin, Mukaila Raji, Bruce Leonard, Lin-Na Chou, Yong-Fang Kuo

**Affiliations:** 1 Department of Obstetrics & Gynecology, The University of Texas Medical Branch at Galveston, Galveston, Texas, United States of America; 2 Center for Interdisciplinary Research in Women’s Health, The University of Texas Medical Branch at Galveston, Galveston, Texas, United States of America; 3 Department of Preventive Medicine and Population Health, The University of Texas Medical Branch at Galveston, Galveston, Texas, United States of America; 4 Department of Internal Medicine, The University of Texas Medical Branch at Galveston, Galveston, Texas, United States of America; 5 Sealy Center on Aging, The University of Texas Medical Branch at Galveston, Galveston, Texas, United States of America; 6 School of Nursing, The University of Texas Medical Branch at Galveston, Galveston, Texas, United States of America; 7 Institute for Translational Science, The University of Texas Medical Branch at Galveston, Galveston, Texas, United States of America; University of South Carolina College of Pharmacy, UNITED STATES

## Abstract

**Background:**

Team care improves processes and outcomes of care, especially for patients with complex medical conditions that require coordination of care. This study aimed to compare the processes and outcomes of care provided to older patients with diabetes by primary care teams comprised of only primary care physicians (PCPs) versus team care that included nurse practitioners (NPs) or physician assistants (PAs).

**Methods:**

We studied 3,524 primary care practices identified via social network analysis and 306,741 patients ≥66 years old diagnosed with diabetes in or before 2015 in Medicare data. Guideline-recommended diabetes care included eye examination, hemoglobin A1c test, and nephropathy monitoring. High-risk medications were based on recommendations from the American Geriatrics Society Beers Criteria for Potentially Inappropriate Medication Use in Older Adults. Preventable hospitalizations were defined as hospitalizations for a potentially preventable condition.

**Results:**

Compared with patients in the PCP only teams, patients in the team care practices with NPs or PAs received more guideline-recommended diabetes care (annual eye exam: adjusted odds ratio (aOR): 1.04 (95% CI: 1.00–1.08), 1.08 (95% CI: 1.03–1.13), and 1.10 (95% CI: 1.05–1.15), and HbA1C test: aOR: 1.11 (95% CI: 1.04–1.18), 1.11 (95% CI: 1.02–1.20), and 1.15 (95% CI: 1.06–1.25) for PCP/NP, PCP/NP/PA, and PCP/PA teams). Patients in the PCP/NP and the PCP/PA teams had a slightly higher likelihood of being prescribed high-risk medications (aOR: 1.03 (95% CI: 1.00–1.07), and 1.06 (95% CI: 1.02–1.11), respectively). The likelihood of preventable hospitalizations was similar among patients cared for by various types of practices.

**Conclusion:**

The team care practices with NPs or PAs were associated with better adherence to clinical practice guideline recommendations for diabetes compared to PCP only practices. Both practices had similar outcomes. Further efforts are needed to explore new and cost-effective team-based care delivery models that improve process, outcomes, and continuity of care, as well as patient care experiences.

## Introduction

Diabetes mellitus has become epidemic and poses a great challenge to the economy and public health in the US [1]. Diabetes incidence is expected to increase in the future, a reflection of the increasing prevalence of obesity and sedentary lifestyles in the US [1]. Long-term complications of diabetes include reduced life expectancy, coronary death, stroke, blindness, kidney failure, and amputation [2]. Over 20% of adults ≥65 years lived with diabetes in 2016 [1]. Optimal primary care access and continuity are especially important for achieving guideline-recommended care [3] in older patients with diabetes, a population at higher risk of complications from diabetes, polypharmacy, and adverse drug events. These risks reflect at least in part the age-related decline in drug metabolism and the use of multiple concomitant medications to manage the multiple comorbid diseases found in this population [4]. Due to primary care physician (PCP) shortage, more primary care for patients with diabetes is being delivered by nurse practitioners (NPs) and physician assistants (PAs), most of whom work with PCPs in a team care model to manage clinically-complex conditions such as diabetes mellitus in older patients [5].

Published evidence shows that the team care model improves processes and outcomes of care, especially for patients with complex medical conditions like diabetes mellitus and heart failure; these conditions often require coordination of care for multiple encounters with various health care providers [5, 6]. Findings from randomized control trials comparing an NP/PCP team care model versus the traditional PCP only model generally showed evidence of increased patient satisfaction, increased adherence, and better clinical outcomes associated with team care [7, 8]. However, all of these studies were conducted in highly selected populations. To get real world evidence, we previously performed an observational study to compare quality of care provided by PCPs only or NPs only vs. a shared care model involving both [9]. The main limitation of this study was that we did not know the extent to which the share care model was actually shared care. In the current cross-sectional study, we used social network analysis (SNA) to identify team practices [10] and assess how the processes and quality of care differ for primary care provided by different types of practices. We assessed guideline-recommended processes of care (e.g., annual eye examination, hemoglobin A1c (HbA1c) test, and nephropathy monitoring) and outcomes of care (e.g., hospitalizations and emergency department [ER] visits) for primary care provided to older patients with diabetes by practices with PCPs only vs. team care with NPs or PAs.

## Materials and methods

### Data source

We first used Medicare claims from providers in 20% randomly selected primary care service areas (PCSAs, n = 1,400) in 2015 to identify primary care practices. Then, we used Medicare claims for beneficiaries diagnosed with diabetes in or before 2015 based on the Chronic Conditions Data Warehouse (CCW) diabetes algorithm to study processes and outcomes of care in 2015. The files included beneficiary summary files, Medicare Provider Analysis and Review (MedPAR) files, Outpatient Standard Analytical Files (OutSAFs), Medicare Carrier files, and Prescription Drug Event records. The 2015 Medicare Shared Savings Program (MSSP) Accountable Care Organization (ACO) provider file was used to identify the ACO affiliation of the primary care practices. The aforementioned data files were located at the CMS Virtual Research Data Center (VRDC). This study was approved by the University of Texas Medical Branch at Galveston Institutional Review Board (IRB # 16–0205). Additionally, a Data Use Agreement was established with the CMS prior to all data analysis.

### Primary care practice identification via social network analysis

We identified primary care providers who worked in the same practice by SNA of Medicare data for all beneficiaries residing at each PCSA [10]. In brief, in each PCSA, we first identified pairs of primary care providers who shared at least 30 patients and then used the Walktrap community finding algorithm to identify clusters of providers [11, 12]. A previous study showed sharing at least 30 patients had 72.2% of positive predictive value in identify primary care provider pairs [10]. Only clusters in PCSAs with a modularity greater than or equal to 0.4 were selected for further analyses. Such clusters will be referred to as primary care practices throughout this paper. Modularity is a measure for clustering and a high modularity indicates dense connections [13]. A modularity equal to or larger than 0.4 is a clear indication that the identified clusters are well defined modules within the network inside the PCSA [14]. Primary care providers were identified using Medicare and Medicaid Services (CMS) provider specialty codes including general practitioners (01), family physicians (08), general internists (11), geriatricians (38), NPs (50), and PAs (97). For NPs and PAs, we further used taxonomy codes (**S1 Table in** S1 Appendix) to identify those in primary care. Two providers were defined as sharing a patient if they both submitted Medicare claims for primary care visits (**S2 Table in** S1 Appendix) for that patient.

### Study cohort

We selected patients with diabetes with at least two office visits to any of the 4,648 practices identified through SNA. Office visits were identified using Current Procedural Terminology (CPT) codes 99201–99205 and 99211–99215. We excluded those cared for by multiple practices and selected patients with complete Medicare parts A, B, and D enrollment without Medicare Advantage enrollment in 2014 and 2015. We then selected patients who were alive through the end of 2015 and aged 66 or older as of January 1, 2015. We excluded residents in long-term-care nursing facilities and those with unknown rural/urban residential information. The long-term-care nursing facility use was identified using OutSAF or carrier claims with CPT codes 99304–99310, 99315, 99316, or 99318 after excluding bills from skilled nursing facilities.

### Study outcome

#### Processes of care

We followed Healthcare Effectiveness Data and Information Set (HEDIS) 2015 measures for comprehensive diabetes care, including eye examination, hemoglobin A1c (HbA1c) test, and nephropathy monitoring [15]. The visits or consultations to endocrinologists, cardiologists, and nephrologists were identified by CPT codes 99201–99205, 99211–99215, or 99241–99245. The number of outpatient office visits to any provider, any primary care provider, and the providers in patients’ own primary care practices were identified by CPT codes 99201–99205 and 99211–99215. We measured three types of continuity of care: all providers, any primary care provider, and providers in patients’ own primary care practices, using the Modified Continuity Index [16].

#### Medication management

We calculated the proportion of days covered by any antidiabetic medications in 2015. For those diagnosed in 2015, the days before the initial diagnosis were not included in the denominator. We followed 2016 HEDIS NDC lists [17] to identify the use of statins, angiotensin-converting enzyme (ACE) inhibitor, angiotensin receptor blocker (ARB), and any high-risk medication in the elderly. The list of high-risk medications (**S3 Table in** S1 Appendix) was selected by the National Committee for Quality Assurance based on recommendations from the American Geriatrics Society Beers Criteria for Potentially Inappropriate Medication Use in Older Adults [18].

#### Inpatient utilization outcomes

Inpatient utilization outcomes included at least one ED visit and preventable hospitalization in 2015. ED visits were identified by any MedPAR claim with a positive ED charge amount or OutSAF claim with a revenue code 0450–0459 or 0981. Preventable hospitalizations were defined by AHRQ as hospitalizations for a potentially preventable condition [19]. Preventable hospitalizations were identified using AHRQ Quality Indicators Software versions 6.0 (ICD-9 and ICD-10).

### Covariates

#### Patient characteristics

Age, gender, race/ethnicity, Medicare-Medicaid dual eligibility, and CCW chronic conditions (including hypertension, hyperlipidemia, ischemic heart disease, arthritis, atrial fibrillation, congestive heart failure, cancer, chronic obstructive pulmonary disease, osteoporosis, chronic kidney disease, depression, asthma, Alzheimer’s disease/dementia, and stroke) were obtained from the beneficiary summary file. The codes used to identify diabetes complications and uncontrolled diabetes are summarized in the **S4 Table** in S1 Appendix. The rurality of the beneficiary’s residence location was determined using the 2013 rural-urban continuum codes from the United States Department of Agriculture [20].

#### Practice characteristics

The NP state regulation variable was classified into three levels: 1) full autonomous practice and prescriptive authority, 2) full autonomous practice and prescriptive authority but requiring a period of supervision, collaboration, or mentorship, and 3) requirement of physician supervision, delegation, consultation, or collaboration [21]. The ACO affiliation of the practices was determined by linking the providers in the practices to the MSSP ACO provider file.

### Statistical analysis

To assess the difference among these four types of practices for each patient and practice characteristic, we calculated the maximum absolute standardized mean difference, which is the maximum of the absolute standardized mean difference between all pairwise comparisons derived from the four types of practices. Generally, a standardized difference of 0.1 or greater is considered a potentially meaningful difference among groups [22]. In our study cohort, patients were clustered in practices. To account for this within-practice correlation, we constructed generalized linear mixed models adjusted for patient characteristics to assess the association between the type of practice and the study outcomes. For eye examination, HbA1c test, nephropathy monitoring, specialist visits/consultation, any use of statins, ACE inhibitors/ARB, high-risk medication, ED visit, and preventable hospitalization, binomial distribution and logit link function were used. The number of outpatient office visits, continuity of care, and proportion of days covered by antidiabetic medications were modeled with normal distribution and identity link function. For each study outcome, we calculated the intraclass correlation coefficient (ICC) which represented the proportion of variance attributed to the practices. SNA was performed using the igraph package in R. All other analyses were performed using SAS Enterprise Guide version 7.15 at the CMS VRDC (SAS Inc., Cary, NC).

## Results

Among the 1,400 PCSAs randomly selected initially, 982 had provider pairs that shared at least 30 patients. After the SNA, 5,284 primary care practices were identified in 559 PCSAs which had a modularity greater than 0.4. We excluded practices with only one provider and then selected these four types of practices: (1) PCP only, (2) PCP and NP, (3) PCP, NP, and PA, and (4) PCP and PA, leaving 4,776 practices. A provider may work in multiple PCSAs and therefore may appear in multiple practices. To diminish this cross-classification, we excluded practices with providers working in multiple practices, resulting in 4,648 practices. Lastly, we selected patients cared for by practices with at least 20 diabetic patients, leaving 306,741 patients and 3,524 practices in the study cohort. The final study cohort included 306,741 patients with diabetes in the US in 2015. They received care from 3,524 practices, including 1,402 PCP only, 1,178 PCP/NP, 432 PCP/NP/PA, and 512 PCP/PA practices (S5 Table in S1 Appendix). Table 1 shows the characteristics of the four types of primary care practices and the characteristics of patients with diabetes cared for by these practices. Patients with diabetes in the PCP only group were more likely to be minorities, dual eligible, and to reside in a metropolitan area. On the other hand, patients in the PCP/NP/PA group were more likely to be non-Hispanic Whites and to reside in an urban or rural area. The prevalence of diabetes complications, uncontrolled diabetes, and comorbidities were similar across patients cared for by these four types of practices. Except for race/ethnicity, dual eligible, and residential area, all standardized differences of patient characteristics across types of primary care practice were less than 0.1.

**Table 1:** Patient and practice characteristics in the four types of primary care practices identified through SNA.

	PCP	PCP/NP	PCP/NP/PA	PCP/PA	Maximum Absolute Standardized Mean Difference^§^
**Patient characteristics**					
Number of patients	107,569	96,731	61,003	41,438	
Age as of Jan 1, 2015, Mean ± SD	75.1 ± 6.7	75.1 ± 6.7	75.1 ± 6.7	75.1 ± 6.7	0.004
Sex, %					
Male	43.8	44.5	45.9	45.5	0.042
Female	56.2	55.5	54.1	54.5	
Race/ethnicity, %					
Non-Hispanic white	73.2	82.5	85.6	83.0	0.348
Black	10.6	9.5	6.8	7.0	
Hispanic	9.2	4.6	4.8	6.3	
Other	6.9	3.4	2.8	3.7	
Dual eligible, %	22.9	18.2	18.0	16.7	0.155
DM complication/uncontrolled DM, %					
Controlled, no complications	42.4	42.8	42.0	43.0	0.068
Controlled, w/ complications	25.0	26.1	26.2	26.9	
Uncontrolled, no complications	11.8	11.0	11.0	10.8	
Uncontrolled, w/ complications	20.9	20.2	20.8	19.3	
Hypertension, %	87.3	87.7	86.5	86.2	0.045
Congestive heart failure, %	21.4	21.3	20.8	19.9	0.037
Ischemic heart disease, %	43.4	43.1	43.3	43.0	0.008
Atrial fibrillation, %	12.2	12.9	13.8	12.7	0.048
Hyperlipidemia, %	76.7	76.8	76.4	77.4	0.025
Stroke, %	4.9	4.8	4.6	4.8	0.016
Arthritis, %	41.2	41.4	39.5	40.6	0.039
Asthma, %	7.1	6.8	6.6	6.7	0.023
Cancer^#^, %	10.8	11.0	10.8	11.0	0.007
Chronic kidney disease, %	35.2	35.0	35.6	34.3	0.029
COPD, %	14.2	15.4	15.6	14.2	0.038
Alzheimer’s disease/dementia, %	9.7	9.6	9.0	8.6	0.038
Depression, %	16.4	17.8	17.5	16.7	0.036
Osteoporosis, %	6.4	5.9	5.7	6.4	0.029
Residence location, %					
Metropolitan	87.2	77.5	75.2	80.7	0.310
Urban	11.7	20.8	22.6	17.9	
Rural	1.1	1.8	2.2	1.3	
**Practice characteristics**					
Number of practices	1,402	1,178	432	512	
State regulation on NP practice/prescribing, %					
Full authority	8.5	8.1	14.6	12.3	0.278
Full authority, conditional	7.1	7.4	11.1	9.0	
Requires physician supervision/collaboration	84.5	84.6	74.3	78.7	
Region of the practice, %					
Midwest	26.3	31.2	22.9	26.2	0.311
Northeast	18.5	19.9	23.8	21.5	
South	34.2	39.4	36.1	34.2	
West	20.9	9.5	17.1	18.2	
MSSP ACO affiliation, %					
All providers^†^	23.5	22.9	0.5	0.8	1.025
None	61.8	57.1	53.9	64.3	
Other	14.7	19.9	45.6	35.0	

ACO: Accountable care organization; COPD: Chronic obstructive pulmonary disease; DM: Diabetes mellitus; MSSP: Medicare shared savings program; NP: Nurse practitioner; PA: Physician assistant; PCP: Primary care physician; SNA: Social network analysis.

^§^A standardized difference of 0.1 or greater is considered a meaningful difference among groups.

^#^Cancer included colorectal, prostate, lung, endometrial, and female breast cancer.

^†^All providers were affiliated with the same ACO.

The PCP/NP/PA group had the highest percentage of full authority or conditional full authority of NP practice/prescribing given by their state regulations. Compared with other types of practices, the PCP/NP practices were more likely to be in the Midwest or South. Most of the practices with PAs did not have all of their providers in the practice affiliated with the same ACO. In contrast to patient characteristics, the standardized differences in practice characteristics were all greater than 0.1.

Processes of care by the type of primary care practice are illustrated in Table 2. Compared with patients in the PCP only group, patients in other practice groups received more guideline-recommended diabetes care, including eye examination, HbA1c test, and nephropathy monitoring, except that the odds ratio for nephropathy monitoring in the PCP/NP group was not significant. The ICCs (**S6 Table in** S1 Appendix) show that more than 10% of the variation in HbA1c testing was attributable to the practices and the finding was similar for nephropathy monitoring. However, the practice-level variation only took up 3.93% of the total variation in eye examination. Patients in the PCP/NP/PA or PCP/PA group received less specialist care from the endocrinologists, while patients in the PCP/NP or PCP/NP/PA group received less specialist care from cardiologists. For specialist visits or consultation, the variation attributable to practices ranged from 8.85% to 17.0%. The number of visits to any provider was the lowest in patients in the PCP/NP group and the number of visits to any primary care provider was the highest in patients in the PCP/NP/PA group. Patients cared for by PCPs only had the highest continuity of care and those in the PCP/NP/PA group had the lowest, regardless of the continuity of care measure examined. For all three measures, the practice-level variations were greater than 10%. Patients in those four groups had similar proportions of days covered by antidiabetics (Table 3). Compared with patients in the PCP only group, patients in the PCP/NP group had a slightly higher likelihood of being prescribed high-risk medications (adjusted odds ratio 1.03, 95% CI 1.00–1.07), as did patients in the PCP/PA group (adjusted odds ratio 1.06, 95% CI 1.02–1.11). Patients in the PCP/NP group had a slightly lower likelihood of being prescribed statins (adjusted odds ratio 0.96, 95% CI 0.93–1.00). For measures related to medication management, the proportions of practice-level variation were not high, ranging from 1.16% to 3.84%.

**Table 2:** Processes of care by the type of primary care practice identified through SNA.

Process of care	PCP	PCP/NP	PCP/NP/PA	PCP/PA	PCP	PCP/NP	PCP/NP/PA	PCP/PA
	%				Odds Ratio (95% CI)			
Diabetes mellitus care^#^								
Eye examination	64.9	65.9	67.9	67.5	1.00	**1.04 (1.00, 1.08)**	**1.08 (1.03, 1.13)**	**1.10 (1.05, 1.15)**
Glycosylated hemoglobin test	91.1	91.6	91.6	92.3	1.00	**1.11 (1.04, 1.18)**	**1.11 (1.02, 1.20)**	**1.15 (1.06, 1.25)**
Monitoring nephropathy	86.7	87.1	87.9	87.0	1.00	1.03 (0.98, 1.09)	**1.17 (1.08, 1.26)**	**1.13 (1.05, 1.22)**
Specialist visits/consultation								
Endocrinologist	10.2	10	9.4	9.1	1.00	0.97 (0.90, 1.04)	**0.87 (0.78, 0.96)**	**0.81 (0.74, 0.90)**
Cardiologist	39.7	39.0	38.2	38.9	1.00	**0.94 (0.90, 0.99)**	**0.88 (0.82, 0.95)**	0.94 (0.88, 1.01)
Nephrologist	8.7	8.7	9.4	8.3	1.00	1.04 (0.97, 1.11)	1.08 (0.99, 1.17)	1.02 (0.93, 1.11)
Number of visits^‡^	Mean ± SD				Adjusted Mean (95% CI)			
To any provider	12.3 ± 8.6	11.8 ± 8.2	12.1 ± 8.3	12.2 ± 8.4	12.2 (12.1, 12.3)	**11.8 (11.7, 12.0)**	12.0 (11.8, 12.2)	12.0 (11.8, 12.2)
To any primary care provider	6.0 ± 4.2	6.0 ± 4.1	6.3 ± 4.3	6.1 ± 4.1	6.0 (5.9, 6.0)	6.0 (6.0, 6.1)	**6.3 (6.2, 6.4)**	**6.2 (6.1, 6.3)**
To the patient’s primary care practice	4.7 ± 3.3	4.5 ± 3.1	4.6 ± 3.2	4.6 ± 3.2	4.6 (4.5, 4.6)	4.5 (4.4, 4.6)	4.5 (4.4, 4.7)	4.6 (4.5, 4.7)
Continuity of care^$^ (range 0–1)								
Any provider	0.64 ± 0.19	0.61 ± 0.19	0.59 ± 0.20	0.62 ± 0.19	0.63 (0.63, 0.63)	**0.61 (0.60, 0.61)**	**0.59 (0.58, 0.59)**	**0.61 (0.61, 0.62)**
Any primary care provider	0.81 ± 0.23	0.77 ± 0.24	0.74 ± 0.25	0.78 ± 0.23	0.80 (0.80, 0.81)	**0.77 (0.76, 0.77)**	**0.72 (0.71, 0.73)**	**0.77 (0.76, 0.78)**
Providers in patient ’s primary care practice	0.90 ± 0.21	0.87 ± 0.23	0.82 ± 0.27	0.87 ± 0.23	0.90 (0.89, 0.90)	**0.87 (0.86, 0.88)**	**0.81 (0.80, 0.82)**	**0.87 (0.86, 0.88)**

CI: Confidence interval; NP: Nurse practitioner; PA: Physician assistant; PCP: Primary care physician; SNA: Social network analysis.

^#^Followed HEDIS 2015 measures for comprehensive diabetes care.

^‡^Adjusted means were estimated using generalized linear mixed models with a negative binomial distribution and a log link function.

^$^The Modified Continuity Index is based on the number of providers and number of visits. It measures the dispersion of care among providers. Index values range from 0 (each visit made to a different physician) to 1 (all visits made to a single physician). Adjusted means were estimated using linear mixed models with normal distribution.

Significant results are in bold, with the PCP practice being the reference group.

**Table 3:** Medication management by the type of primary care practice identified through SNA.

Medication management	PCP	PCP/NP	PCP/NP/PA	PCP/PA	PCP	PCP/NP	PCP/NP/PA	PCP/PA
	Mean ± SD				Adjusted Mean (95% CI)			
Proportion of days covered by antidiabetics^‡^	79.0 ± 27.2	79.5 ± 27.1	79.5 ± 27.0	79.6 ± 27.0	79.1 (78.8, 79.3)	79.4 (79.2, 79.7)	79.3 (78.9, 79.7)	79.4 (79.0, 79.8)
	%				Odds Ratio (95% CI)			
Use of statin^#^	73.1	72.3	73.3	72.5	1.00	**0.96 (0.93, 1.00)**	1.01 (0.96, 1.06)	0.99 (0.94, 1.04)
Angiotensin-converting enzyme inhibitor or angiotensin receptor blocker^#^^$^	75.1	74.9	75.1	75.3	1.00	0.99 (0.96, 1.02)	1.01 (0.97, 1.05)	1.03 (0.98, 1.07)
Use of high-risk medication^#^	19.2	20.4	19.7	20.3	1.00	**1.03 (1.00, 1.07)**	1.00 (0.96, 1.05)	**1.06 (1.02, 1.11)**

CI: Confidence interval; NP: Nurse practitioner; PA: Physician assistant; PCP: Primary care physician; SD: standard deviation; SNA: Social network analysis.

^‡^Patients who did not have any antidiabetic prescription in 2014 and 2015 were excluded, leaving 225,202 patients. Adjusted means were estimated using linear mixed models with normal distribution.

^#^Followed HEDIS 2016 NDC lists.

^$^The corresponding results were produced in patients with hypertension (N = 267,239).

Significant results are in bold, with the PCP practice being the reference group.

Inpatient utilization outcome measures by the four types of primary care practices are presented in Table 4. Patients in those four types of practices had similar rates of preventable hospitalizations. The only notable difference was that patients in the PCP/NP/PA group had more ER visits than patients in the PCP only group (adjusted odds ratio 1.06, 95% CI 1.01–1.10). The variation attributable to practices were only 2.92% for any emergency department visits and 3.47% for any preventable hospitalization. Instead of adjust for CCW chronic conditions, we repeated our analyses adjusted for Charlson comorbidity index, our conclusions remained the same, except for patients in the PCP/NP group also had more ER visits than patients in the PCP only group (adjusted odds ratio 1.03, 95% CI (1.01–1.07)).

**Table 4:** Outcome of care by the type of primary care practice identified through SNA.

Outcome of care	PCP	PCP/NP	PCP/NP/PA	PCP/PA	PCP/NP vs. PCP	PCP/NP/PA vs. PCP	PCP/PA vs. PCP
	%				Odds Ratio (95% CI)		
Any ED visit	36.7	37.4	37.2	35.5	1.02 (0.99, 1.06)	**1.06 (1.01, 1.10)**	0.98 (0.93, 1.02)
Any preventable hospitalization^#^	5.1	5.2	5.1	4.7	0.98 (0.92, 1.03)	1.00 (0.93, 1.07)	0.96 (0.89, 1.03)

CI: Confidence interval; ED: Emergency department; NP: Nurse practitioner; PA: Physician assistant; PCP: Primary care physician; SNA: Social network analysis.

^#^Preventable hospitalizations were identified using AHRQ Quality Indicators Software versions 6.0.1 (designed for International Classification of Diseases, Tenth Revision, Clinical Modification [ICD-10-CM] codes) and 6.0.2 (designed for International Classification of Diseases, Ninth Revision, Clinical Modification [ICD-9-CM] codes).

## Discussion

Using Medicare claims in 2015, we assessed processes and outcomes of care for primary care provided to older patients with diabetes by different types of practice (PCP only vs. team care with NPs or PAs). We found that patients with diabetes cared for by team care with NPs or PAs received more guideline-recommended diabetes care, including eye examination, HbA1c testing, and nephropathy monitoring. However, they were also slightly more likely to be prescribed high-risk medications. Overall, patients cared for by PCPs only and those cared for by team care with NPs or PAs had similar outcomes, such as preventable hospitalizations and ER visits, except that patients in the PCP/NP/PA group were more likely to have ER visits.

Past studies show that use of a team-based care model for patients with diabetes is associated with higher odds of better adherence to clinical practice guidelines vis-a-vis the process of diabetes care [23]. A recent systemic review also found that patients receiving nurse practitioner-physician co-management were more likely to follow recommended care guidelines and reach practice and clinical targets [24]. Our findings also showed better adherence to diabetes care recommendations in patients cared for by team care with NPs or PAs compared with patients cared for by PCPs only. Most of the diabetes care measures were also better than those we previously reported in older patients cared for by NPs only [25]. However, we do not know how much of this difference was due to the improvement in care over time. Patients with diabetes cared for by team care with NPs or PAs received more eye examinations, HbA1c testing, and nephropathy monitoring, as recommended by clinical practice guidelines. Team-based and patient-centered care delivery models facilitate greater teamwork, better coordination and integration of care, effective communication, and partnership with patients, which may also improve the patient care experience [26, 27]. The American Diabetes Association “Standards of Medical Care in Diabetes” promotes care systems that facilitate team-based care and recommends that diabetes care should be managed by a multidisciplinary team, composed of PCPs, subspecialty physicians, NPs, PAs, and other health professionals [28].

Overall, the outcomes in terms of preventable hospitalizations and ER visits were comparable in patients cared for by PCPs only versus those cared for by team care with NPs or PAs. Our previous studies in older patients with diabetes cared for by NPs only or PCPs only also reported similar rates of preventable hospitalization [25, 29]. Other studies generally reported comparable or slightly improved clinical outcomes in patients with diabetes when NPs or PAs are involved in primary care delivery [30, 31]. An integrative literature review of the effectiveness of a patient-centered medical home model also failed to prove the model’s efficacy in relation to diabetes clinical quality outcomes such as HbA1c, blood pressure, and blood lipids, although some of the reviewed studies demonstrated improvements in one or two of those outcome measures [23]. A recent systemic review found variability in clinical patient outcomes, with some findings favoring nurse practitioner-physician co-management and limited differences in clinical quality outcomes [24]. Additional efforts are needed to identify new and cost-effective team-based care delivery models that improve patient care experiences, process, outcomes, and continuity of care.

Continuity of care is associated with increased patient satisfaction [32]. As expected, the lower level of continuity of care in patients cared for by team care with NPs or PAs likely reflects the patients’ inability to always be seen by the same provider at every visit over time, as we found in our study. However, Moira Stewart proposed that this might be a legitimate chicken-or-egg discussion regarding continuity and satisfaction [33], since it is possible that continuity of care may just be a proxy for a satisfying patient-physician relationship. In that scenario, satisfied patients will insist on seeing the same provider, while dissatisfied patients may be more likely to change providers over time. This is an interesting area for future study.

We also observed a slightly higher likelihood of high-risk medication prescription in the PCP/NP and the PCP/PA practices. More NPs and PAs practice in a rural setting [34], which may partly explain the observed higher prescription of high-risk medications, as opportunities for frequent NP/MD and PA/MD clinical collaborations diminish. However, the PCP/NP/PA group did not show an increased prescription of high-risk medications, and they had the highest percentage of rural residents. This finding may reflect the increased opportunities for NP/PA clinical collaborations when unclear prescribing questions arise. Nevertheless, further study is needed to confirm the presence of and clarify the underlying reasons for high-risk medication prescription in older patients with diabetes in team practices.

The main strength of this study is the broad coverage of our data source to include all Medicare diabetes patients meeting selection criteria and our use of SNA of data to identify different types of primary care practices in the 20% randomly selected primary care service areas. This methodology allowed us to comprehensively study processes, outcomes, and continuity of care nationally across different models of practices, with a focus on the outcomes that matter most to patients.

This study had several limitations. The findings from Medicare patients may not be generalizable to patients covered by commercial insurance or younger patients. In addition, we relied on the diagnosis codes on the claims to determine the diabetes complication and whether or not the diabetes condition was controlled. We do not have access to laboratory results (e.g., blood glucose levels HbA1c) to assess how well patients’ diabetes was actually controlled. Additionally, we did not examine temporal patterns in processes and outcomes of care across primary care models. Longitudinal studies are needed to characterize the variations in care delivery and patient outcomes across primary care models. Furthermore, claims data do not have information on the roles of NPs and PAs while providing services. We used SNA to identify providers working closely together. However, it is unknown whether NPs and PAs acted as a substitute for a PCP or contributed their complementary skills to patient care in a true team care fashion.

## Conclusions

Team care practices with NPs or PAs tend to follow the clinical practice guideline recommendations for diabetes care better compared to PCP only practices. Patients in team care practices had lower continuity of care and fewer specialist consultations. Their chronic medication management and outcomes were similar to those of PCP only patients. Slightly higher high-risk medication use was found in the PCP/NP and PCP/PA practices. Overall, the variation in continuity of care and specialist consultations across practices were relatively large compared to medication management and outcomes of care.

## Supporting information

S1 Appendix (DOCX)
