# Supplementary material for: Processes and outcomes of diabetes mellitus care by different types of team primary care models
Source: PLoS One. 2020 Nov 5;15(11):e0241516. doi: 10.1371/journal.pone.0241516 (PMC7644045; doi:10.1371/journal.pone.0241516)
Supplement: S1 Appendix — (DOCX) [file pone.0241516.s001.docx]

S1 Table. Taxonomy codes for identifying NPs and PAs in primary care.

| Taxonomy code | Description |
| --- | --- |
| 363L00000X | Nurse Practitioner |
| 363LA2200X | NP-Adult Health |
| 363LF0000X | NP-Family |
| 363LG0600X | NP-Gerontology |
| 363LP2300X | NP-Primary Care |
| 363LW0102X | NP-Women's Health |
| 363A00000X | Physician Assistant |
| 363AM0700X | PA-Medical |

S2 Table. Codes used for identifying primary care visits.

| CPT/HCPCS code | Description |
| --- | --- |
| 99201-99215 | Office or Other Outpatient Services |
| 99304-99318 | Nursing Facility Services |
| 99324-99337 | Domiciliary, Rest Home (eg, Boarding Home), or Custodial Care Services |
| 99339-99340 | Domiciliary, Rest Home (eg, Assisted Living Facility), or Home Care Plan Oversight Services |
| 99341-99350 | Home Services |
| 99363-99368 | Case Management Services |
| 99374-99380 | Care Plan Oversight Services |
| 99381-99429 | Preventive Medicine Services |
| 99441-99449 | Non-Face-to-Face Physician Services |
| 98966-98969 | Non-Face-to-Face Nonphysician Services |
| 99487-99490 | Care Management Services |
| 99495-99496 | Transitional Care Management Services |
| 99497-99498 | Advanced Care Planning |
| G0463 | Hospital outpatient clinic visit |
| G0438-G0439 | Annual wellness visit |

S3 Table. High-Risk Medications.

| Type A: Any Prescription |  | |
| --- | --- | --- |
| Description | Prescription | |
| Anticholinergics (excludes TCAs), first-generation antihistamines | - Brompheniramine - Carbinoxamine - Chlorpheniramine - Clemastine - Cyproheptadine - Dexbrompheniramine | - Dexchlorpheniramine - Diphenhydramine (oral) - Doxylamine - Hydroxyzine - Promethazine - Triprolidine |
| Anticholinergics (excludes TCAs), anti-Parkinson agents | - Benztropine (oral) | - Trihexyphenidyl |
| Antithrombotics | - Dipyridamole, oral short-acting (does not apply to the extended-release combination with aspirin) | - Ticlopidine |
| Cardiovascular, alpha agonists, central | - Guanabenz - Guanfacine | - Methyldopa |
| Cardiovascular, other | - Disopyramide | - Nifedipine, immediate release |
| Central nervous system, tertiary TCAs | - Amitriptyline - Clomipramine | - Imipramine - Trimipramine |
| Central nervous system, barbiturates | - Amobarbital - Butabarbital - Butalbital - Mephobarbital | - Pentobarbital - Phenobarbital - Secobarbital |
| Central nervous system, vasodilators | - Ergot mesylates | - Isoxsuprine |
| Central nervous system, other | - Thioridazine - Chloral Hydrate | - Meprobamate |
| Endocrine system, estrogens with or without progestins; include only oral and topical patch products | - Conjugated estrogen - Esterified estrogen | - Estradiol - Estropipate |
| Endocrine system, sulfonylureas, long-duration | - Chlorpropamide | - Glyburide |
| Endocrine system, other | - Desiccated thyroid | - Megestrol |
| Gastrointestinal system, other | - Trimethobenzamide |  |
| Pain medications, skeletal muscle relaxants | - Carisoprodol - Chlorzoxazone - Cyclobenzaprine | - Metaxalone - Methocarbamol - Orphenadrine |
| Pain medications, other | - Indomethacin - Ketorolac, includes parenteral | - Meperidine - Pentazocine |
| **Type B: Days of Supply >90 days** |  |  |
| Anti-Infectives, other | - Nitrofurantoin - Nitrofurantoin macrocrystals | - Nitrofurantoin macrocrystals-monohydrate |
| Nonbenzodiazepine hypnotics | - Eszopiclone - Zaleplon | - Zolpidem |
| **Type C: With Average Daily Dose Criteria** |  |  |
| Description | Prescription | Average Daily Dose Criteria |
| Alpha agonists, central | - Reserpine | >0.1 mg/day |
| Cardiovascular, other | - Digoxin | >0.125 mg/day |
| Tertiary TCAs (as single agent or as part of combination products) | - Doxepin | >6 mg/day |

S4 Table. Codes used for identifying diabetes complications and uncontrolled diabetes.

| Description | ICD-9-CM | ICD-10-CM |
| --- | --- | --- |
| Uncontrolled diabetes mellitus | 249.01, 249.11, 249.21, 249.31, 249.41, 249.51, 249.61, 249.71, 249.81, 249.91, 250.02, 250.03, 250.12, 250.13, 250.22, 250.23, 250.32, 250.33, 250.42, 250.43, 250.52, 250.53, 250.62, 250.63, 250.72, 250.73, 250.82, 250.83, 250.92, 250.93 | E08.65, E10.65, E11.65 |
| Complications of diabetes mellitus (renal manifestations, ophthalmic manifestations, neurologic manifestations, peripheral circulatory disorders, other specified manifestations and unspecified manifestations) | 249.4X, 249.5X, 249.6X, 249.7X, 249.8X, 249.9X, 250.4X, 250.5X, 250.6X, 250.7X, 250.8X, 250.9X, 357.2, 362.01, 362.02, 362.03, 362.04, 362.05, 362.06, 366.41 | E08.21 ‑ E08.29, E08.311 ‑ E08.39, E08.40 ‑ E08.49, E08.51 ‑ E08.59, E08.610 - E08.649, E08.69, E08.8 E10.21 ‑ E10.29, E10.311 ‑ E10.39, E10.40 ‑ E10.49, E10.51 ‑ E10.59, E10.610 - E10.649, E10.69, E10.8 E11.21 ‑ E11.29, E11.311 ‑ E11.39, E11.40 ‑ E11.49, E11.51 ‑ E11.59, E11.610 - E11.649, E11.69, E11.8 |

S5 Table. Cohort selection.

|  | N, patient | %, prior step | N, practice | %, prior step |
| --- | --- | --- | --- | --- |
| 1. Select diabetic* patients with at least 2 office visits to any of the **4,648** practices identified through SNA. | 678,965 |  | 4,465 |  |
| 2. Exclude those cared for by multiple practices | 645,358 | 95.1 | 4,464 | 100.0 |
| 3. Select patients with complete parts A, B, and D enrollment without Medicare Advantage enrollment in 2014 and 2015. | 431,064 | 66.8 | 4,458 | 99.9 |
| 4. Select patients alive on 12/31/2015. | 429,010 | 99.5 | 4,458 | 100.0 |
| 5. Select patients aged 66^$^ or above | 342,189 | 79.8 | 4,447 | 99.8 |
| 6. Remove residents in long-term-care nursing facilities | 316,709 | 92.6 | 4,437 | 99.8 |
| 7. Remove those with unknown rural/urban residential information | 316,514 | 99.9 | 4,437 | 100.0 |
| 8. Select patients cared for by practices with at least 20 patients | 306,741 | 96.9 | 3,524 | 79.4 |

*Based on the Chronic Condition Warehouse (CCW) end-of-year flag.

^$^As of Jan 1, 2015.

S6 Table. Intraclass correlation coefficient (ICC) for primary care practices identified through SNA

|  | ICC (%) |
| --- | --- |
| Process of care |  |
| Diabetes mellitus care |  |
| Eye examination | 3.93 |
| Glycosylated hemoglobin test | 10.82 |
| Monitoring nephropathy | 9.44 |
| Specialist visits/consultation |  |
| Endocrinologist | 17.00 |
| Cardiologist | 8.85 |
| Nephrologist | 10.99 |
| Number of visits |  |
| To any provider | 9.00 |
| To any primary care provider | 12.37 |
| To the pt's primary care practice | 14.06 |
| Continuity of care |  |
| Any provider | 11.00 |
| Any primary care provider | 13.95 |
| Providers in pt's primary care practice | 16.53 |
|  |  |
| Medication management |  |
| Proportion of days covered by antidiabetics | 1.16 |
| Use of statin | 3.84 |
| Angiotensin-converting enzyme inhibitor or angiotensin receptor blocker | 1.93 |
| Use of high-risk medication | 2.15 |
|  |  |
| Outcome of care |  |
| Any ED visit | 2.92 |
| Any preventable hospitalization | 3.47 |
